# Fenton and Photo-Fenton Degradation of Chlorpyrifos Using α-Mn_2_O_3_ Heterogeneous Catalysis

**DOI:** 10.3390/ijms27135856

**Published:** 2026-06-29

**Authors:** Silviu-Laurentiu Badea, Violeta-Carolina Niculescu, Marian-Nicolae Verziu, Teodor-Adi Ene, Liliana-Aurelia Badulescu

**Affiliations:** 1 Research Center for Studies of Food Quality and Agricultural Products, University of Agronomic Sciences and Veterinary Medicine of Bucharest, 59 Marasti Blvd., 011464 Bucharest, Romania; liliana.badulescu@qlab.usamv.ro; 2 National Research and Development Institute for Cryogenic and Isotopic Technologies—ICSI Rm. Vâlcea, 4th Uzinei Street, 240050 Ramnicu Vâlcea, Romania; violeta.niculescu@icsi.ro (V.-C.N.); teodor.ene@icsi.ro (T.-A.E.); 3 Department of Bioresources and Polymer Science, National Research Center for Micro and Nanomaterials, National University of Science and Technology Politehnica Bucharest, 313 Spl. Independenței, 060042 Bucharest, Romania; marianverziu32@gmail.com; 4 Department of Materials Science and Engineering, Faculty of Materials and Environmental Engineering, Technical University of Cluj-Napoca, 400641 Cluj-Napoca, Romania

**Keywords:** chlorpyrifos, manganese trioxide, Fenton, photo-Fenton

## Abstract

Chlorpyrifos, a widely used organophosphate pesticide, poses significant environmental risks due to its persistence and the formation of toxic transformation products. Despite extensive research on iron-based Fenton systems, the application of manganese oxides, particularly α-Mn_2_O_3_, in chlorpyrifos degradation remains insufficiently explored. In this study, we investigated the catalytic performance of α-Mn_2_O_3_ in Fenton and visible-light-driven photo-Fenton processes for the degradation of chlorpyrifos in aqueous systems. Chlorpyrifos oxon was identified as a transient intermediate, detected at trace levels, supporting an oxidative degradation pathway. Kinetic analysis revealed pseudo-first-order behavior, with comparable rate constants for Fenton reactions at different catalyst loadings (0.0033 min^−1^ for 5 mg and 0.0028 ± 0.0006 min^−1^ for 10 mg), indicating that the process is not limited by catalyst concentration under the investigated conditions. In contrast, the photo-Fenton system exhibited a higher rate constant (0.0042 min^−1^) and significantly improved degradation efficiency, highlighting the role of visible-light activation. The highest removal rates of chlorpyrifos were 86.24% for Fenton experiments and 96.05% for the photo-Fenton experiment, respectively. The enhanced performance is attributed to the photocatalytic properties of α-Mn_2_O_3_, including its narrow bandgap and the facilitation of Mn^3+^/Mn^2+^ redox cycling, which promotes reactive oxygen species generation. These findings demonstrate that α-Mn_2_O_3_ is a promising non-iron catalyst for advanced oxidation processes and provide new insights into manganese-mediated Fenton-like mechanisms for the removal of organophosphate contaminants.

## 1. Introduction

Chlorpyrifos is a synthetic insecticide (specifically an organophosphate) that has been widely used in agriculture to control insects on crops like corn, soybeans, fruit trees, and vegetables. Regarding its insecticidal properties, chlorpyrifos (CPF) acts by irreversibly inhibiting acetylcholinesterase, causing toxic accumulation of acetylcholine and resulting in widespread nervous system overstimulation [1,2]. Because of its toxicity and persistence, chlorpyrifos was banned in the EU (in 2020) and the USA (in 2024), while in 2025 it was listed under the Stockholm Convention on Persistent Organic Pollutants.

Chlorpyrifos occurrence in the aquatic environment can harm aquatic organisms like invertebrates, fish and plants [3], since, due to its extensive usage in agriculture, it has been detected at various levels in water bodies. Although less persistent than organochlorines, chlorpyrifos can persist in freshwater bodies and soils, with half-lives varying from hours to months [4,5]. Furthermore, degradation of chlorpyrifos in the aquatic environment can produce degradation products like chlorpyrifos-oxon, which has higher toxicity compared to the parent compound CPF [6]. The formation of chlorpyrifos-oxon was also shown to occur as a by-product of drinking water chlorination [7].

Advanced oxidation processes (AOPs) [8,9] can be defined as chemical processes that generate in situ reactive radicals like hydroxyl (·OH with shorter half-lives of 20 ns) or sulfate (SO_4_· with longer half-lives of 30–40 µs) [10] for the oxidative transformation of organic pollutants [11,12,13]. One key characteristic of AOPs is that they can effectively eliminate organic compounds (like dyes, pesticides and pharmaceuticals) in the aqueous phase rather than accumulating or transferring pollutants into another phase, as other treatment techniques do (like adsorption, filtration, precipitation, flocculation, etc.). The ·OH radicals are among the most powerful known oxidants and can effectively break down a wide range of organic pollutants into harmless compounds, such as CO_2_, water, nitrogen and chloride and bromide ions for halogenated organic contaminants [14]. Besides the nature of the involved radicals (hydroxyl (·OH) or sulfate (SO_4_·)), AOPs can be subdivided into homogeneous or heterogeneous processes [9]. Homogeneous AOPs have been used extensively in the last 20 years to remove organic contaminants from wastewater, also using different forms of energy (ultrasound, UV/visible radiation, or electrochemical) [15]. With respect to chlorpyrifos, various degradation rates have been reported for homogeneous Fenton degradations. Gandhi et al. [16] recorded a value of 6.8 × 10^−4^ min^−1^ for the degradation rate constant of CPF, while after 240 min of reaction time, the removal rate of chlorpyrifos by Fenton reaction was just 15.1%, using 5 mg/L Fe^2+^ and 100 mg/L H_2_O_2_. In contrast to the above-mentioned study, the authors of [17] found that at a molar ratio H_2_O_2_/Fe^2+^ equal to 10 and a molar ratio H_2_O_2_/chemical oxygen demand (COD) of 3 (initial pH 3), a complete degradation of chlorpyrifos occurred after one minute. Saini et al. [18] developed an optimization methodology for the Fenton oxidation of CPF and found that at concentrations of 0.571 mol/L for H_2_O_2_ and 3 g/L for Fe^2+^ (pH of 3), the best CPF removal efficiency of 94% was obtained after 60 min of reaction. Regarding photo-Fenton degradation studies of CPF, this process is considered more efficient than the classical Fenton process [19]. For example, Gandhi et al. [16] recorded a degradation rate constant of 3.3 × 10^−4^ min^−1^ for chlorpyrifos during photo-Fenton degradation with a 400 W UV lamp (with wavelength ranged from 200 to 400 nm), while the removal rate of CPF was about 50.3% after 30 min of UV exposure, also using similar conditions of 5 mg/L Fe^2+^ and 100 mg/L H_2_O_2_ as for the Fenton reaction. In another study, Murillo et al. [20] investigated the photo-Fenton degradation of CFP and using optimized conditions (the concentrations were 10 mg/L FeCl_3_, 0.01 M of H_2_O_2_, at an initial pH value of 3.5), a complete degradation of chlorpyrifos was recorded after 15 min of degradation using the light of a solar chamber. Chlorpyrifos-oxon was the main degradation product of chlorpyrifos, detected after 10 min of degradation in a concentration of less than 80 µg/L [20]. Regarding the heterogeneous photo-Fenton degradation of chlorpyrifos, in the same study mentioned above [20], a complete degradation of CPF was obtained after 20 min when 10 mg/L TiO_2_ were used in the solar chamber as a photo-catalyst at a concentration of H_2_O_2_ of 0.02 M.

Also, with respect to the heterogeneous photo-Fenton using non-iron catalysts, manganese oxides have been used in recent years as Fenton catalyst because of the excellent redox characteristics of Mn ions and their high stability and reusability at pH values higher than 5.5 where no dissolution occurs [21,22]. Very recently, a combination of MnO_2_ and Mn_2_O_3_ were used to perform the advanced oxidation of unsymmetrical dimethylhydrazine (UDMH) in a microwave-assisted reaction [23]. Among them, manganese(III) oxide, especially as the bixbyite phase of α-Mn_2_O_3_ with cubic structure, has been shown to catalyze Fenton reactions for many organic pollutants [21]. Nevertheless, there are very few studies on the use of α-Mn_2_O_3_ as a catalyst in (photo-)Fenton studies on organophosphate pesticides.

The objective of this paper was to investigate Fenton and photo-Fenton degradation of chlorpyrifos using α-Mn_2_O_3_ obtained by green laboratory synthesis, using citric acid and potassium permanganate. This study is one of the initial investigations into the green synthesis of α-Mn_2_O_3_ for visible-light-assisted Fenton degradation of chlorpyrifos with chlorpyrifos-oxon tracking. Moreover, the influence of the amount of α-Mn_2_O_3_ as well as the influence of visible light on the catalysis process were investigated.

## 2. Results

### 2.1. Characterization of α-Mn_2_O_3_ and Relationship with Its Catalytic Properties

**Figure 1:**
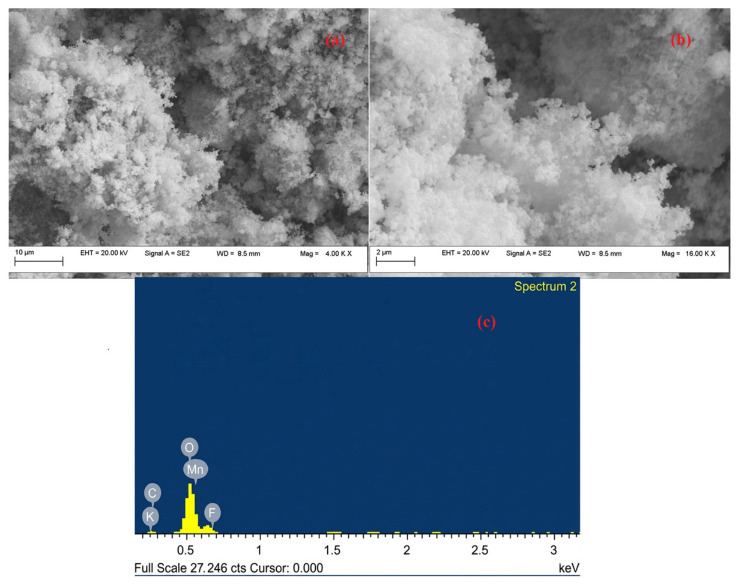
SEM images of the α-Mn_2_O_3_ nanoparticles recorded at different magnification levels: 10 µm (**a**) and 2 µm (**b**); EDX spectrum recorded at 70 µm (**c**).

The α-Mn_2_O_3_ batches used in the three experiments were characterized by SEM in order to evaluate their morphology and surface characteristics. The SEM micrographs revealed the presence of agglomerated α-Mn_2_O_3_ nanoparticles, with some particles exhibiting a quasi-spherical morphology. Representative SEM images recorded at different magnification levels are presented in Figure 1a,b.

The SEM-EDX analysis confirmed that manganese and oxygen were the predominant elements in the synthesized nanoparticles (Figure 1c). The obtained atomic percentages were 58.58% for Mn and 30.80% for O, while the corresponding weight percentages were 30.31% and 54.72%, respectively (see Supplementary Materials, Table S1). These values are consistent with those reported in the literature for laboratory-synthesized Mn_2_O_3_ materials [22,23]. The observed morphology, characterized by nanoparticle agglomeration and irregular surface structure, may contribute to the catalytic performance of α-Mn_2_O_3_ by providing accessible active sites and facilitating surface interactions during the catalytic degradation process [24,25].

**Figure 2:**
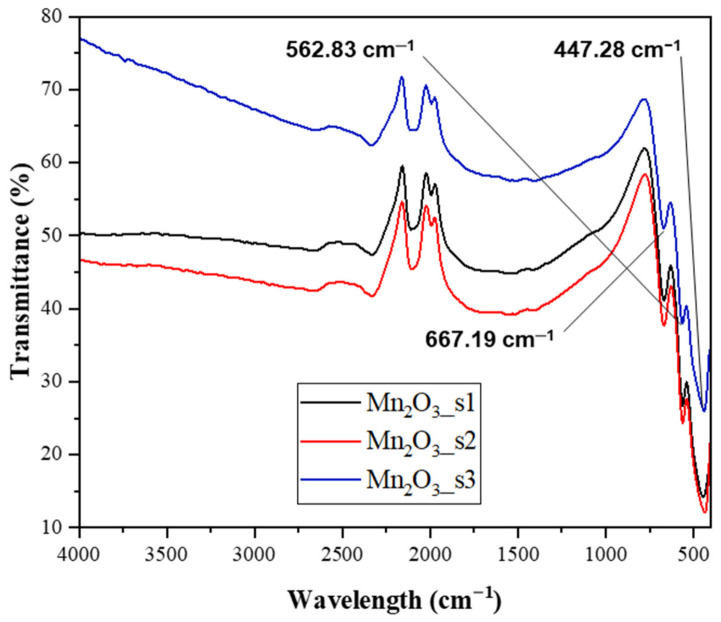
FTIR spectra of the synthesized α-Mn_2_O_3_ catalyst obtained from triplicate measurements.

The FTIR spectra (Figure 2) exhibited highly reproducible features across the three independently recorded samples, indicating good consistency of both the catalyst synthesis and spectroscopic measurements. The observed characteristic bands corresponded mainly to vibrational modes associated with Mn–O bonds in the manganese oxide lattice, together with minor contributions arising from surface-adsorbed species [26,27,28,29,30,31]. The most intense spectral features occurred in the low-wavenumber region (400–700 cm^−1^), typical for metal–oxygen vibrations in transition metal oxides. Three main bands were identified near 441, 563, and 668 cm^−1^, corresponding to Mn–O lattice vibrations of the MnO_6_ octahedral units forming the manganese oxide structure. Similar spectral features have been widely reported for Mn_2_O_3_ and related manganese oxides [26,27,29,30,31]. According to Julien et al. [29], bands located between 400–500 cm^−1^ are typically associated with Mn–O bending or lattice deformation modes, while bands observed near 520–650 cm^−1^ correspond to Mn–O stretching vibrations within MnO_6_ octahedra. Therefore, the presence of these bands confirmed the successful formation of a manganese oxide framework consistent with α-Mn_2_O_3_. Recent studies on Mn_2_O_3_ nanostructures have reported similar FTIR bands around 520–620 cm^−1^, attributed to Mn–O and Mn–O–Mn vibrations within the oxide lattice [28,32]. Consequently, the band detected near 562.8 cm^−1^ was considered a diagnostic feature of the Mn_2_O_3_ structure.

In addition to lattice vibrations, some weak bands were detected in the mid-infrared region. A band around 1513 cm^−1^ was assigned to carbonate species formed by the adsorption of atmospheric CO_2_ on the oxide surface. Such carbonate species commonly form on metal oxide catalysts through the interaction between CO_2_ and surface oxygen atoms or hydroxyl groups, particularly when the surface exhibits basic or defect sites [33]. Another weak band centered near 1552 cm^−1^ was attributed to the bending vibration of adsorbed water molecules (H–O–H deformation mode). Surface hydration may frequently be observed in transition metal oxides due to the presence of unsaturated metal centers able to interact with atmospheric moisture [33]. The relatively low intensity of these bands indicated that the amount of adsorbed species was limited and did not significantly affect the structural characteristics of the oxide phase. To quantitatively evaluate the reproducibility of the FTIR measurements, the peak positions were extracted from the three spectra and averaged. The resulting values, expressed as mean ± standard deviation, are summarized in Table 1.

The relatively small standard deviation values confirmed the excellent reproducibility of the FTIR spectra, mainly for the characteristic Mn–O stretching band at 562.8 cm^−1^, which exhibited identical peak positions in all three measurements. This consistency supports the reliability of the catalyst preparation method and indicates a stable manganese oxide structure. More importantly, the spectra did not exhibit significant absorption bands in regions typically associated with organic functional groups, such as C–H stretching vibrations (2850–2950 cm^−1^) or C=O stretching bands (~1700 cm^−1^) [27,28]. The absence of these signals suggested that the precursor species were effectively removed during the thermal treatment step, resulting in the formation of a predominantly inorganic oxide phase. Similar observations were reported for calcined Mn_2_O_3_ catalysts and nanomaterials, where complete decomposition of precursor compounds leads to clean Mn–O spectral signatures [27,28].

Overall, the FTIR analysis confirmed the formation of a manganese oxide lattice characterized by Mn–O vibrations typical for α-Mn_2_O_3_. The presence of minor bands associated with adsorbed water and carbonate species reflected the interaction of the oxide surface with atmospheric components after synthesis; this phenomenon is commonly observed for transition metal oxide catalysts [33,34].

**Figure 3:**
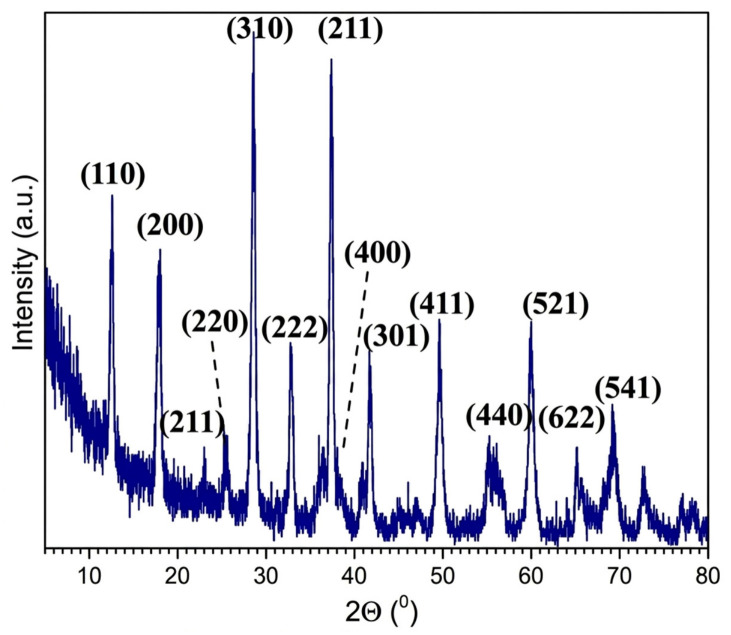
X-ray diffraction pattern of the synthesized Mn_2_O_3_.

The XRD pattern highlighted the presence of both cubic α-Mn_2_O_3_ and tetragonal α-MnO_2_ phases (Figure 3). The XRD pattern highlighted the presence of both tetragonal α-MnO_2_ and cubic α-Mn_2_O_3_ phases. The diffraction angles (2Ɵ) noted at approximately 23.1°, 32.9°, 38.2°, 55.1° and 65.7° correspond to the (211), (222), (400), (440) and (622) planes of cubic α-Mn_2_O_3_ [35]. However, the diffraction angles (2Ɵ) of 12.7°, 18.1°, 28.7°, 37.4°, 49.8°, 60.3° and 69.7° can be assigned to the (110), (200), (310), (121), (411), (521) and (451) crystallographic planes of tetragonal α-MnO_2_ [36].

Moreover, the partial overlap of diffraction angles in the regions around 37–38°, 49–50°, 55–56° and 65–66° further suggests the coexistence of both manganese oxide phases.

### 2.2. Degradation of Chlorpyrifos by the Fenton Process

**Figure 4:**
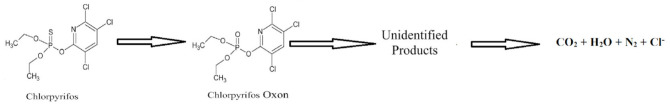
Degradation pathway of chlorpyrifos via chlorpyrifos oxon in the Fenton H_2_O_2/_Mn_2_O_3_ system. Figure modified from [37].

Chlorpyrifos was degraded in experiments lasting up to 6 h (360 min). In Experiments 1 and 3, diethyl (3,5,6-trichloropyridin-2-yl) phosphate (chlorpyrifos oxon) was detected as the main intermediate degradation product (see Supplementary Materials, Figures S2 and S3). However, the chlorpyrifos oxon was detected only in trace quantities, and no reliable concentration of this intermediate could be determined. The other major oxic degradation product of CPF is 3,5,6-trichloro-2-pyridinol (TCP), as suggested in previous studies [7]. Nevertheless, in the current study, no TCP was detected. Based on chlorpyrifos oxon as an intermediate degradation product, this supports previously proposed degradation pathways of CPF in AOP reactions [37], including in the Fenton H_2_O_2_/Mn_2_O_3_ system from this paper (Figure 4).

**Figure 5:**
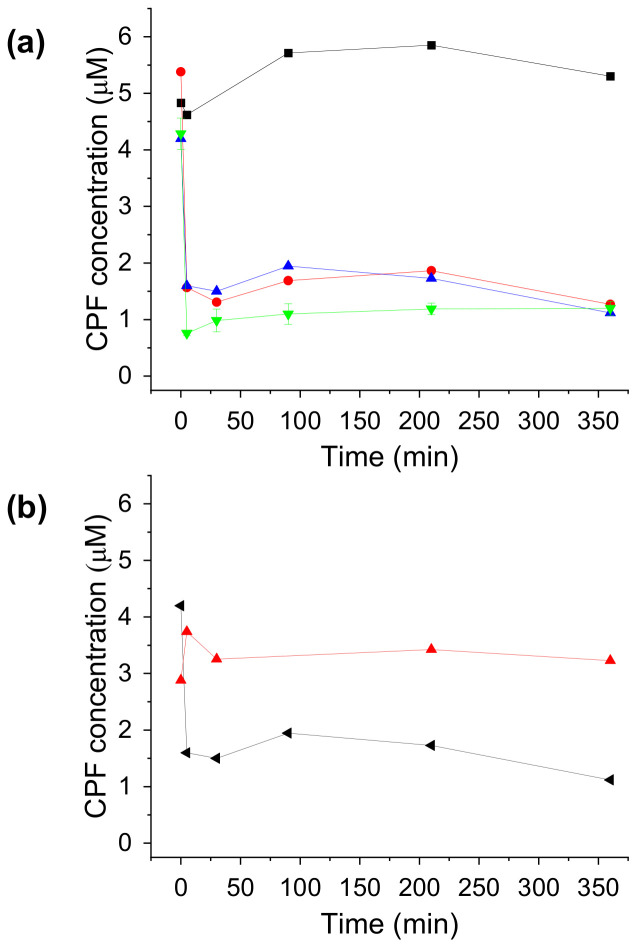
Degradation of CPF in the 1st Fenton experiment in the presence of 0 mg (●), 5 mg (▲) and 10 mg (▼) of α**-**Mn_2_O_3_ and 421.2 mM H_2_O_2_. The evolution of CPF concentrations in the control bottle (■) (**a**). Degradation of CPF in two Fenton experiments with 5 mg of α**-**Mn_2_O_3_ using different concentrations of H_2_O_2_: 144.5 mM (▲) and 421.2 mM (◄) (**b**).

In the 1st experiment, CPF was transformed in all three bottles where the Fenton reaction was performed. In the 1st bottle (without α-Mn_2_O_3_), the concentration of CPF decreased from 5.38 µM at the beginning of the 1st experiment to 1.57 µM after 5 min of degradation and finally to 1.27 µM after 360 min, at the end of the experiment (Figure 5a). In the 2nd bottle (with 5 mg of α-Mn_2_O_3_), the concentration of CPF decreased from 4.20 µM at the start of Experiment 1 to 1.60 µM after 5 min of degradation and finally to 1.12 µM after 360 min, at the end of the experiment. In the 3rd bottle (10 mg of α-Mn_2_O_3_ in duplicates), the concentration of CPF decreased from 4.29 ± 0.28 µM at the beginning of Experiment 1 to 0.76 ± 0.04 µM after 5 min of degradation and finally to 1.20 ± 0.12 µM after 360 min, at the end of the experiment. Thus in this experiment, the highest removal rate of CPF was 86.24% for the 10 mg α-Mn_2_O_3_ bottle. These concentration values from bottles 2 and 3 of the 1st experiment indicate that the amount of α-Mn_2_O_3_ catalyst added in this current Fenton system does not influence the degradation of chlorpyrifos. In the control bottle, CPF concentration varied from 4.83 μM at the start of the Fenton reaction to 5.30 μM after 360 min, at the end of Experiment 1, showing no significant transformation of CPF, while no degradation products were detected.

In the 2nd experiment, due to the low initial concentration of H_2_O_2_ (144.5 mM) in the system, the degradation of CPF was small, its concentration evolving from 2.88 µM at the beginning of the experiment to 3.23 µM, showing no clear trend (Figure 5b). In contrast, in the 1st experiment, CPF showed a clear degradation trend at the same amount of 5 mg α-Mn_2_O_3_. In the 3rd experiment, the degradation of CPF was very fast due to the high concentration of H_2_O_2_ (889.2 mM), and no degradation kinetics could be established (see Supplementary Materials, Figure S1).

### 2.3. Degradation of Chlorpyrifos by Photo-Fenton Process

**Figure 6:**
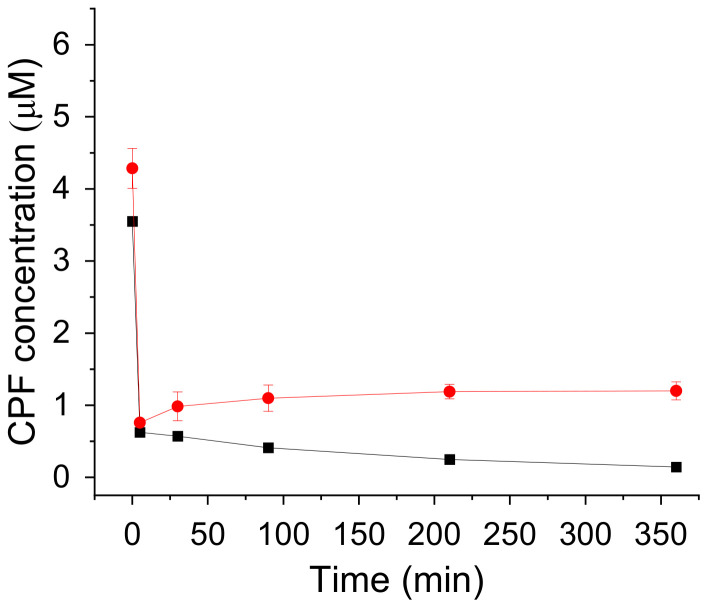
Degradation of CPF in Fenton (●) and photo-Fenton (■) experiments using 10 mg α-Mn_2_O_3_ and 421.2 mM H_2_O_2_.

In order to demonstrate the influence of visible light on the Fenton reaction, a photo-Fenton experiment was performed, and the decrease in CPF concentration was compared with that of the Fenton degradation in bottle 3 of Experiment 1 using the same reagent concentrations (10 mg of α-Mn_2_O_3_, 421.2 mM H_2_O_2_). This comparison is presented in Figure 6. The decrease in CPF concentration after 5 min of degradation was similar for both Fenton and photo-Fenton reactions: from 4.29 ± 0.28 µM at the beginning of the experiment to 0.76 ± 0.04 µM for Fenton and from 3.55 µM to 0.63 µM for photo-Fenton, respectively. Nevertheless, after 360 min of degradation, the CPF concentration was 1.20 ± 0.12 µM for the Fenton reaction and 0.14 µM for the photo-Fenton reaction, showing that photo-Fenton degradation is much more efficient (96.05% removal efficiency) compared with Fenton degradation, as suggested in previous studies [20].

## 3. Discussion

Using the concentration data obtained from different experiments, the degradation rate constants (k) of CPF were calculated (Table 2) assuming that the dehalogenation of CPF in Fenton and photo-Fenton experiments is driven by pseudo-first-order kinetics.

The k values showed that in the 1st experiment, a value of 0.0033 min^−1^ was recorded for the bottle with 5 mg catalyst, while for the bottle with 10 mg α-Mn_2_O_3_, the recorded k value was 0.0028 ± 0.0006 min^−1^, further confirming no influence of the amount of catalyst.

**Figure 7:**
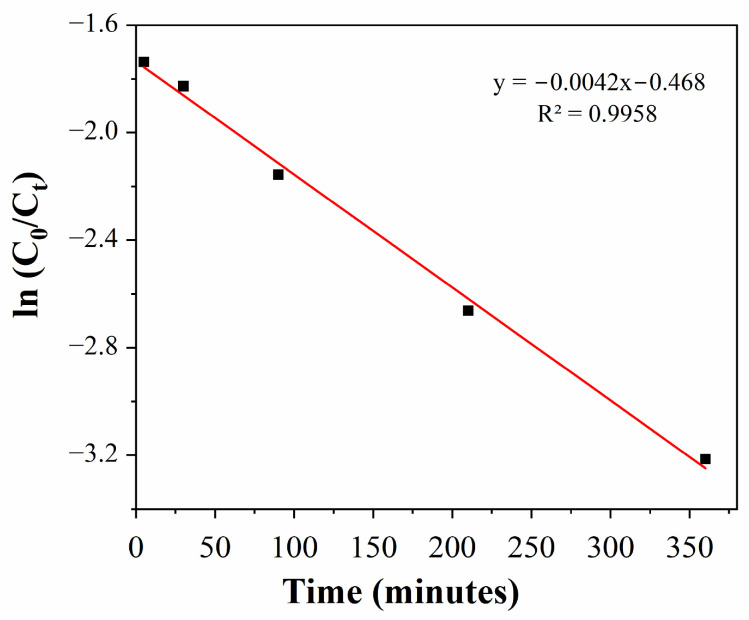
Quantitative assessment of the degradation rate of CPF in the Photo-Fenton experiment.

The k value of 0.0042 min^−1^ recorded for the photo-Fenton experiment is higher compared with the two values mentioned above (see Figure 7), demonstrating the efficiency of the photo-Fenton process in visible light in removing CPF. These k values (0.0033 min^−1^ and 0.0028 ± 0.0006 min^−1^) recorded in the Fenton process from our study are slightly higher than the value of 6.8 × 10^−4^ min^−1^ recorded by Gandhi et al. [16] in a homogeneous Fenton study (5 mg/L Fe^2+^ and 100 mg/L H_2_O_2_). Also, the k value 0.0042 min^−1^ recorded for the photo-Fenton experiment is slightly lower than the k value of 7.8 × 10^−3^ min^−1^ recorded by Gandhi et al. [16] in UV/H_2_O_2_ degradation in the presence of 100 mg/L H_2_O_2_. Furthermore, the k value from the photo-Fenton experiment in our study is on the same order of magnitude as the k value of 0.0036 min^−1^ recorded by Mondal et al. in the UV/H_2_O_2_ system, but lower than the value of 0.0441 min^−1^ recorded in the same very recent study using a UV/H_2_O_2_ system employing Mn/Fe co-doped titanium dioxide nanoparticles conjugated with graphene oxide (GO) as a photocatalyst [38]. This efficiency of the photo-Fenton process from our study is due to the fact that Mn_2_O_3_ is an active photocatalyst under visible light, as demonstrated in recent studies [39]. Its narrow bandgap (ranging between 1.29–1.98 eV) allows it to absorb visible light, making it suitable for degrading organic pollutants, dyes and pharmaceuticals [40]. In this respect, the FTIR analysis (see Figure 2) showed that the Mn–O stretching bands detected in the 520–670 cm^−1^ region reflected the presence of MnO_6_ octahedra within the Mn_2_O_3_ lattice. They are often correlated with lattice defects and oxygen vacancies. These structural features may enhance catalytic activity by increasing oxygen mobility and facilitating reversible Mn^3+^/Mn^2+^ redox cycles [23,41], which are essential for the oxidation and environmental catalytic processes involving manganese oxide catalysts [28,30,34].

## 4. Materials and Methods

### 4.1. Chemical Reagents

Chlorpyrifos (CPF, C_9_H_11_Cl_3_NO_3_PS, 99% analytical purity), hexabromobenzene (HBB, C_6_Br_6_, 98% analytical purity), and hydrogen peroxide (H_2_O_2_, 30 wt.% concentration, 99% purity) were purchased from Sigma-Aldrich (Darmstadt, Germany). Dichloromethane (DCM, CH_2_Cl_2_, 99.8% analytical purity), methanol (CH_3_OH, 99.8% analytical purity), and anhydrous sodium sulfate (Na_2_SO_4_, 99% analytical purity) were acquired from Honeywell (Offenbach am Main, Germany). Chlorpyrifos oxon (C_9_H_11_Cl_3_NO_4_P, 99% analytical purity) was purchased from Dr. Ehrenstorfer (Augsburg, Germany).

### 4.2. Green Synthesis of Manganese (III) Oxide Nanoparticles

Manganese (III) oxide nanoparticles were synthesized by adding a 250 mL solution of 0.3 M citric acid to a 250 mL solution of 0.3 M potassium permanganate. The synthesis experiment was performed over 3 h in a water bath heated by a combined hot-plate magnetic-stirrer device (LLG-Labware, Meckenheim, Germany) at 30 °C and mixed at 1000 rpm. The precipitate was transferred to 50 mL tubes, washed with ethanol and with distilled water between ethanol washings, and then separated by centrifugation at 7500 rpm for 10 min. The precipitate was then dried at 45 °C for 12 h in a drying oven and further converted to α-Mn_2_O_3_ by calcination in an oven (Naberthem, Bremen, Germany) at 500 °C for 24 h.

### 4.3. Characterization of Manganese(III) Oxide Nanoparticles

The manganese (III) oxide nanoparticles were characterized by powder scanning electron microscopy (SEM) coupled with energy-dispersive X-ray spectroscopy (EDS/EDX), FTIR spectroscopy and X-ray diffraction (XRD).

SEM-EDX measurements were recorded using a FESEM SIGMA VP scanning electron microscope (SEM) coupled with an energy-dispersive X-ray spectrometer (EDS) (Carl Zeiss, Oberkochen, Germany). Using these measurements, the morphological characterization of the α-Mn_2_O_3_ nanoparticles was carried out.

FTIR spectroscopy was performed using a Cary 630 ATR-FTIR spectrometer (Agilent Technologies, Santa Clara, CA, USA). Prior to analysis, the solid samples were finely ground using an agate mortar with pestle and subsequently dried under vacuum at 80 °C to remove physically adsorbed moisture. The spectra were recorded over the range of 4000–400 cm^−1^ with a resolution of 8 cm^−1^, 32 scans, and a threshold value of 0.002. Each spectrum represented the average of multiple scans to improve the signal-to-noise ratio. To ensure reproducibility, the measurements were performed in triplicate, and the resulting spectra were superimposed to evaluate the consistency of the spectral features. The spectra were processed using the instrument software MicroLab Expert v1.0.0 (Agilent Technologies, Santa Clara, CA, USA) and the peak positions were extracted from the raw spectral datasets.

X-ray diffraction (XRD) patterns were recorded using a Rigaku Ultima IV diffractometer operating in parallel-beam geometry with Cu Kα radiation (λ = 1.5418 Å) at 40 kV and 30 mA. Data were collected over a 2θ range of 5–80°, with a scan speed of 2°/min and a step size of 0.02°.

### 4.4. Fenton Degradation Experiments

In all experiments, the Fenton degradations were carried out in 0.5 L Schott Duran bottles (18.1 cm height, 4.5 cm screw thread, and 7.8 cm side wide). Chlorpyrifos was added from a stock solution in acetone of 2.79 mM to aqueous solutions of 200 mL, resulting in a theoretical concentration of 5.5 µM, just below its aquatic solubility limit of 5.7 µM (2 mg L^–1^). In the 1st experiment, the oxidation reaction was initiated by adding 9 mL of H_2_O_2_ (30% *w*/*w* concentration, 9.8 M) to all bottles containing the aqueous solution of chlorpyrifos (aquatic concentration of H_2_O_2_: 421.2 mM). The bottles were amended with different amounts of α-Mn_2_O_3_ nanoparticles: no α-Mn_2_O_3_ added in bottle 1 (initial pH of 6.81), 5 mg (bottle 2, initial pH of 6.93), and 10 mg in duplicates (bottle 3, initial pH of 6.190 ± 0.004). In the 2nd experiment, the oxidation reaction was initiated by adding 3 mL of H_2_O_2_ (30% *w*/*w* concentration) to a bottle containing the aqueous solution of chlorpyrifos (aquatic concentration of H_2_O_2_: 144.5 mM) with 5 mg α-Mn_2_O_3_ (initial pH of 6.90). In the 3rd experiment, the oxidation reaction was initiated by adding 20 mL of H_2_O_2_ (30% *w*/*w* concentration) to all five bottles containing the aqueous solution of chlorpyrifos (aquatic concentration of H_2_O_2_: 889.2 mM), with different amounts of α-Mn_2_O_3_ nanoparticles: without α-Mn_2_O_3_ (bottle 1, initial pH of 5.48), 5 mg (bottle 2, initial pH of 5.74), 10 mg in duplicate (bottle 3, initial pH of 5.94 ± 0.04) and 20 mg (initial pH of 6.16). In all experiments, control bottles containing chlorpyrifos spiked in deionized water at the same concentration of 5.5 µM were used. In all experiments, the bottles were shaken on an incubator with orbital shaking (Lab Companion, Jeio Tech, Billerica, MA, USA) at 150 rpm and 30 °C for 3–4 h before the 1st sample was taken, and afterwards at 125 rpm for 6 h. At regular intervals, 10 mL of aqueous samples were taken with plastic syringes for liquid-liquid extraction. Upon sampling, 2 mL of methanol was added to the samples and then mixed with a vortex at 3000 rpm for 1 min to quench the Fenton reaction. The aliquots of chlorpyrifos and its degradation products were extracted in 16 mL vials with 1 mL DCM containing hexabromobenzene (HBB) as an internal standard. The liquid–liquid extraction was performed by mixing the samples with a vortex at 3000 rpm for 1 min, and afterwards for 3 h at 200 rpm on a horizontal shaker.

### 4.5. Photo-Fenton Degradation Experiments

The photo-Fenton reaction was carried out in a glass vessel with an internal diameter of 9.5 cm and a height of 22 cm inserted between a crown of visible light LEDs with a wavelength ranging from 435 to 445 nm (Sigma–Aldrich, Darmstadt, Germany). In total, 10 mg α-Mn_2_O_3_ nanoparticles were added as a photo-catalyst. Chlorpyrifos was added from a stock solution to aqueous solutions of 200 mL (pH of 6.39) at the same theoretical concentration of 5.5 µM. The photo-Fenton reaction was carried out for 6 h on a combined hot-plate magnetic-stirrer device at room temperature, mixed at 700 rpm until the first sample was collected and afterwards at 500 rpm. The photo-oxidation reaction was initiated by adding 9 mL of H_2_O_2_ (30% *w*/*w* concentration, 9.8 M) simultaneously with the start-up of the visible light LEDs (aquatic concentration of H_2_O_2_: 421.2 mM). At regular intervals, 10 mL of aqueous samples were taken with plastic syringes; the sample preparation and extraction of the samples were the same as for the Fenton experiments.

### 4.6. Gas Chromatography–Mass Spectrometry (GC-MS) Analysis

In the 1st Fenton experiment and in the photo-Fenton experiment, chlorpyrifos and its degradation products were determined using an Agilent 7890B Gas Chromatograph (GC) coupled to an Agilent 7010 triple quadrupole Mass Spectrometer (MS, Agilent Technologies, Santa Clara, CA, USA), operated in positive electron ionization (EI) mode (70 eV) and used in SCAN/SIM mode. Samples (1 μL volume) were inserted at 250 °C into the injector, which was operated in split mode, while the flow rate of the carrier gas (He) was 1.2 mL min^–1^. The target compounds were separated on an HP-5MS capillary column (5% Phenyl Polysilphenylene-siloxane, 30 m × 0.25 mm × 0.25 μm, Agilent Technologies, Santa Clara, CA, USA) using the following oven temperature program: initial temperature of 50 °C (5 min isothermal), ramped at 5 °C min^−1^ to 200 °C (0 min), 5 °C min^−1^ to 230 °C (0 min) and 8 °C min^−1^ to 300 °C (3 min isothermal). When operated in SIM mode, the following ions and dwell time settings were used: 197 (80 ms), 314 (80 ms), and 552 (80 ms).

For the 2nd and 3rd Fenton experiments, an Agilent 8890 GC coupled to an Agilent 5977 C inert Mass Selective Detector (Agilent Technology, Santa Clara, CA, USA) was employed. The MS was configured in synchronous selected ion monitoring (SIM)/scan mode, while samples (0.5 μL volume) were inserted at 280 °C into the injector operated in split mode. The target compounds were separated on the same type of HP-5MS GC column (Agilent Technologies, Santa Clara, CA, USA) using the following temperature program: initial temperature of 50 °C (1 min isothermal), ramped at 10 °C min^−1^ to 300 °C (5 min isothermal), with a helium flow rate of 1 mL min^–1^. The ions and dwell time settings for SIM recording were the same as in the above-mentioned SIM method.

To detect trace amounts of chlorpyrifos oxon, the same GC parameters were used in the Agilent 7890B GC (Agilent Technologies, Santa Clara, CA, USA) in splitless injection mode, while the Agilent 7010 triple quadrupole MS was operated in multiple reaction monitoring (MRM) mode. The following *m*/*z* transitions were used in the MS-MS: 298 to 269.9 and 298 to 241.8 for chlorpyrifos oxon, and 552.6 to 473.6 and 552.6 to 392.6 for HBB.

## 5. Conclusions

This study demonstrates that α-Mn_2_O_3_ is an effective heterogeneous catalyst for the degradation of chlorpyrifos in both Fenton and photo-Fenton systems. The results confirm that significant degradation occurs under all investigated conditions, and the detection of chlorpyrifos oxon as a transient intermediate supports an oxidative degradation pathway consistent with advanced oxidation processes.

The kinetic analysis indicates that the apparent degradation rate is not significantly influenced by catalyst loading within the investigated range, suggesting that the reaction is primarily governed by oxidant availability and radical generation rather than by the number of active surface sites. In contrast, the application of visible light irradiation in the photo-Fenton system leads to a clear enhancement of degradation efficiency and reaction kinetics. This behavior highlights the photocatalytic contribution of α-Mn_2_O_3_, which can be attributed to its narrow bandgap and its ability to promote Mn^3+^/Mn^2+^ redox cycling, thereby facilitating the continuous generation of reactive oxygen species.

The combined structural and spectroscopic characterization indicates that the catalytic performance is closely related to the physicochemical properties of the material, including the presence of Mn–O lattice structures and potential defect sites that may enhance oxygen mobility and redox activity. These features are likely responsible for the observed catalytic behavior under both dark and irradiated conditions.

Overall, the findings demonstrate that α-Mn_2_O_3_ represents a promising non-iron catalyst for advanced oxidation processes targeting organophosphate pesticides. The improved performance under visible light further supports its applicability in sustainable and energy-efficient water treatment systems. Future work should focus on the detailed identification of degradation products using high-resolution mass spectrometry (HRMS) techniques and on elucidating the reaction mechanisms at the molecular level in order to better assess the environmental fate and safety of transformation products.

## Figures and Tables

**Table 1:** Characteristic FTIR bands of the synthesized α-Mn_2_O_3_ catalyst with peak positions determined from triplicate spectra (mean ± SD).

Vibrational Band (Assignment)	Spectrum 1 (cm^−1^)	Spectrum 2 (cm^−1^)	Spectrum 3 (cm^−1^)	Mean ± SD (cm^−1^)	Interpretation
Mn–O bending vibration	447.28	436.10	439.83	441.07 ± 5.69	Lattice deformation of MnO_6_ octahedra
Mn–O stretching vibration	562.83	562.83	562.83	562.83 ± 0.00	Characteristic Mn–O vibration of manganese oxide framework
Mn–O lattice vibration	667.19	667.19	670.92	668.44 ± 2.15	Mn–O stretching/Mn–O–Mn lattice vibration
CO_3_^2−^ asymmetric stretching	1513.30	1517.03	1509.57	1513.30 ± 3.73	Surface carbonate species from CO_2_ adsorption
H–O–H bending	1554.30	1554.30	1546.85	1551.81 ± 4.30	Adsorbed water molecules

**Table 2:** The values of the rate constants for the degradation of CPF in Fenton and photo-Fenton experiments.

No.	Amount of α-Mn_2_O_3_	Apparent Rate Constant (min^−1^)	Fenton Experiment
1	5	0.0033	1st
2	10	0.0028 ± 0.0006	1st
3	10	0.0042	Photo-Fenton

## Data Availability

The original contributions presented in this study are included in the article/Supplementary Materials. Further inquiries can be directed to the corresponding author.

## Supplementary Materials

The following supporting information can be downloaded at https://www.mdpi.com/article/10.3390/ijms27135856/s1.
