# Supplementary material for: Fenton and Photo-Fenton Degradation of Chlorpyrifos Using α-Mn2O3 Heterogeneous Catalysis
Source: Int J Mol Sci. 2026 Jun 29;27(13):5856. doi: 10.3390/ijms27135856 (PMC13361091; doi:10.3390/ijms27135856)
Supplement: Supplementary file 1 [file ijms-27-05856-s001.zip › ijms-4333441-supplementary.pdf]

Supplementary Materials for the manuscript
Fenton and Photo-Fenton degradation of chlorpiryfos using α-Mn_2O_3 heterogeneous catalysis

Silviu-Laurentiu Badea[1]*, Violeta-Carolina Niculescu[2], Marian-Nicolae Verziu[3], Teodor-Adi Ene[2,4], Liliana- Aurelia Badulescu[1]

[1]*Research Center for Studies of Food Quality and Agricultural Products, University of Agronomic Sciences and Veterinary Medicine of Bucharest, 59 Marasti Blvd., 011464, Bucharest, Romania (e-mails: silviu.badea@qlab.usamv.ro; liliana.badulescu@qlab.usamv.ro)*

[2]*National Research and Development Institute for Cryogenic and Isotopic Technologies – ICSI Rm. Vâlcea, 4th Uzinei Street, 240050 Ramnicu Vâlcea, Romania (e-mails: violeta.niculescu@icsi.ro, teodor.ene@icsi.ro)*

[3] *Department of Bioresources and Polymer Science, National Research Center for Micro and Nanomaterials, National University of Science and Technology Politehnica Bucharest, 313 Spl. Independenței, 060042 Bucharest, Romania (marianverziu32@gmail.com)*

[4]*Department of Materials Science and Engineering, Faculty of Materials and Environmental Engineering, Technical University of Cluj-Napoca, 400641, Cluj-Napoca, Romania*

*Correspondence: silviu.badea@qlab.usamv.ro

Materials and Methods

Characterization and quantification of CPF and its degradation products by GC-MS-MS

The sample preparation of δ-HCH and its degradation products from aquatic samples was performed in 16 mL vials with 1 mL dichloromethane containing 45 μM hexabromobenzene (HBB) as internal standard. In every GC-MS sequence, a standard solution containing CPF and HBB, was analysed for assessing the reproducibility of the response factors in the GC-MS analyses.

In order to detect the trace amount of chlorpyrifos oxon, the same GC parameters as in the main text were used in the Agilent 7890B gas chromatograph (GC), with the use of splitless injection mode, while the Agilent 7010 triple quadrupole mass spectrometer was operated in Multiple Reaction Monitoring (MRM). The following m/z transitions were used in the MS-MS: 298 → 269.9 and 298 → 241.8 for chlorpyrifos oxon. and 552.6 → 473.6 and 552.6 → 392.6 for HBB.

Results and discussion

Characterization of FeS nanoparticles by SEM-EDS

The EDS analysis was resulting in an EDS spectrum that incdicated the main elements of the characterized nanoparticles as manganise and oxygen. The atomic and weight percents were 30.8% and 54.72 % for O and 58.58 % and 30.31 % for Mn, respectively. The other elements were identified only at trace levels (see table S1).

Table S1. Atomic and weight and percent of major elements identified by EDX in a batch of synthesized α-Mn_2O_3 nanoparticles.

Element/Line	Mn-K	O-K	F-K	K-K	C-K	Totals
Atomic (%)	58.58	30.8	4.15	4.01	2.47	
Weight (%)	30.31	54.72	6.21	2.92	5.85	100

Identification of degradation products of CPF by GC-MS-MS

The chlorpyrifos oxon was identified as the main degradation products of CPF in experiment 1 and 3. The figures S1 is showing a chromatogram recorded after 210 minutes of degradation in experiment no. 3 showing the complete degradation of CPF and the peak of chlorpyrifos oxon at 18.949 minutes, while his mass spectrum is presented in figure S2.

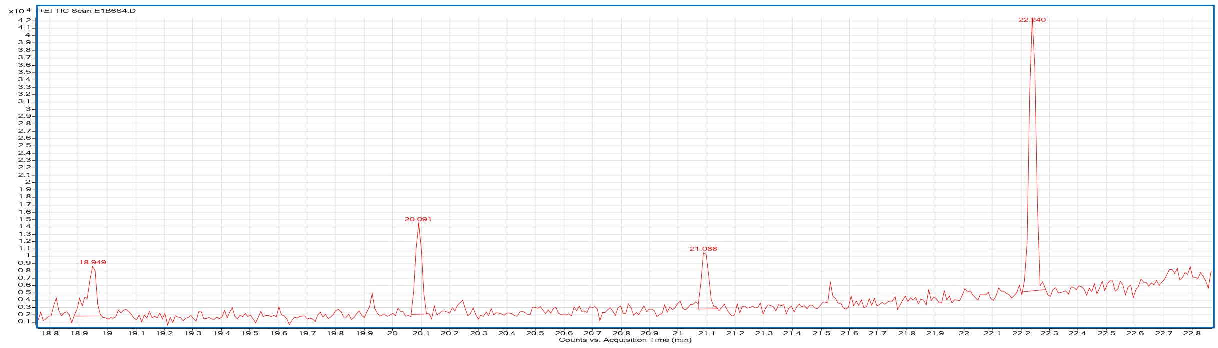

Figure S1. Total Ion Current chromatogram of sample recorded 210 minutes of degradation showing the peaks of chlorpyrifos oxon at 18.949 minutes in the 3rd experiment.

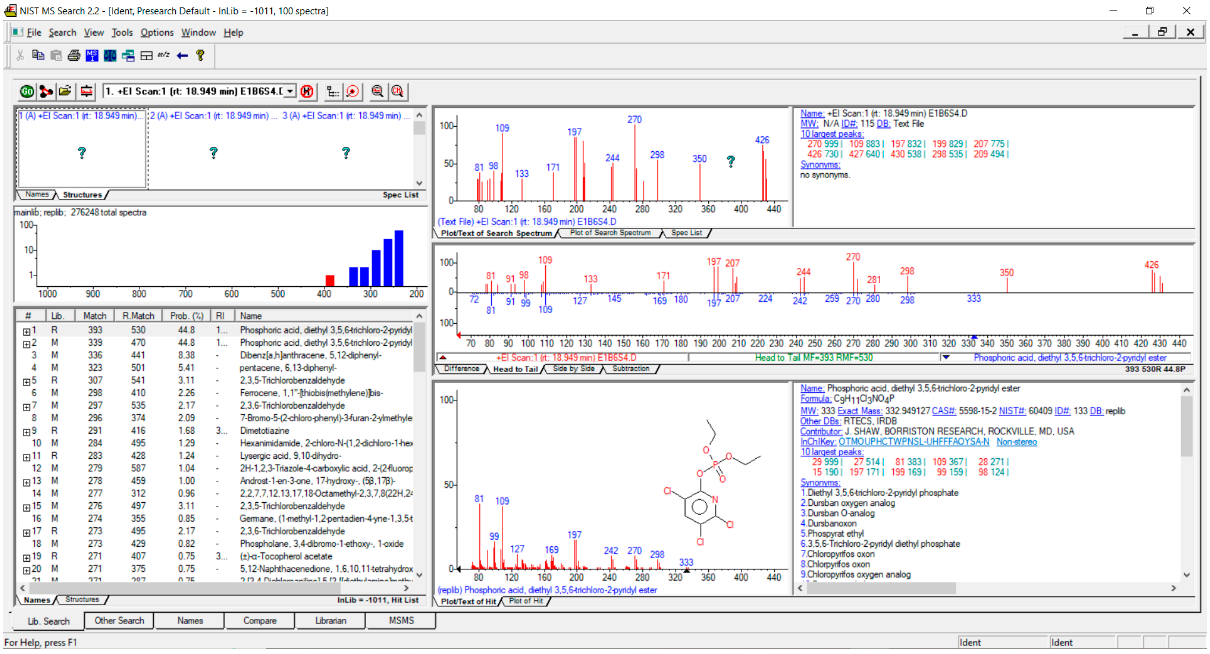

Figure S2. The mass spectrum of chlorpyrifos oxon at 18.949 minutes recorded after 210 minutes in the 3rd experiment.

In the 1st experiment was detected in MRM mode. In figure S3 it is shown an MRM chromatogram of chlorpyrifos oxon at 25.968 minutes recorded after 5 minutes of degradation in the 1st experiment vs MRM chromatogram authentic chlorpyrifos oxon standard of about 21 µg/mL. The height of the chlorpyrifos oxon peak in the sample is about 1000-fold lower comparing with the standard showing that the chlorpyrifos oxon occurred in trace quantities only (levels of ng/mL).

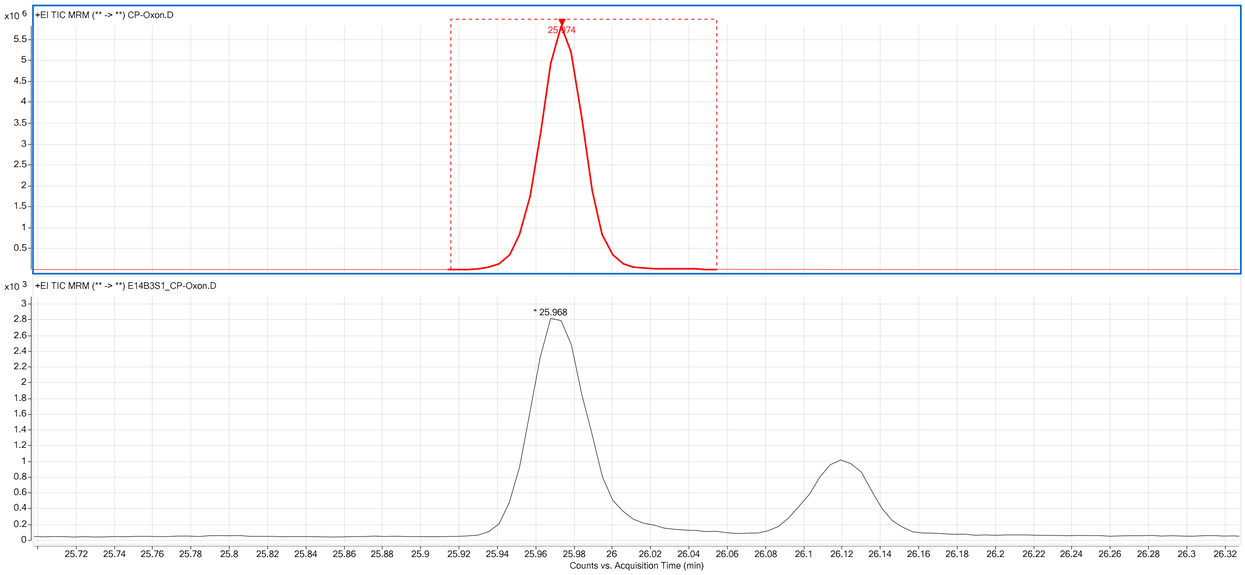

Figure S3. The MRM chromatogram of chlorpyrifos oxon at 25.968 minutes recorded after 5 minutes of degradation in the 1st experiment vs MRM chromatogram authentic chlorpyrifos oxon standard.
